# The Application of Gaussian Mixture Models for Signal Quantification in MALDI-ToF Mass Spectrometry of Peptides

**DOI:** 10.1371/journal.pone.0111016

**Published:** 2014-11-05

**Authors:** John Christian G. Spainhour, Michael G. Janech, John H. Schwacke, Juan Carlos Q. Velez, Viswanathan Ramakrishnan

**Affiliations:** 1 Medical University of South Carolina, Department of Public Health Sciences, Charleston, South Carolina, United States of America; 2 Division of Nephrology, Department of Medicine, Medical University of South Carolina, Charleston, South Carolina, United States of America; 3 Ralph H. Johnson Veterans Affairs Medical Center, Charleston, South Carolina, United States of America; 4 Scientific Research Corporation, North Charleston, South Carolina, United States of America; Max-Delbrück Center for Molecular Medicine (MDC), Germany

## Abstract

Matrix assisted laser desorption/ionization time-of-flight (MALDI-TOF) coupled with stable isotope standards (SIS) has been used to quantify native peptides. This peptide quantification by MALDI-TOF approach has difficulties quantifying samples containing peptides with ion currents in overlapping spectra. In these overlapping spectra the currents sum together, which modify the peak heights and make normal SIS estimation problematic. An approach using Gaussian mixtures based on known physical constants to model the isotopic cluster of a known compound is proposed here. The characteristics of this approach are examined for single and overlapping compounds. The approach is compared to two commonly used SIS quantification methods for single compound, namely Peak Intensity method and Riemann sum area under the curve (AUC) method. For studying the characteristics of the Gaussian mixture method, Angiotensin II, Angiotensin-2-10, and Angiotenisn-1-9 and their associated SIS peptides were used. The findings suggest, Gaussian mixture method has similar characteristics as the two methods compared for estimating the quantity of isolated isotopic clusters for single compounds. All three methods were tested using MALDI-TOF mass spectra collected for peptides of the renin-angiotensin system. The Gaussian mixture method accurately estimated the native to labeled ratio of several isolated angiotensin peptides (5.2% error in ratio estimation) with similar estimation errors to those calculated using peak intensity and Riemann sum AUC methods (5.9% and 7.7%, respectively). For overlapping angiotensin peptides, (where the other two methods are not applicable) the estimation error of the Gaussian mixture was 6.8%, which is within the acceptable range. In summary, for single compounds the Gaussian mixture method is equivalent or marginally superior compared to the existing methods of peptide quantification and is capable of quantifying overlapping (convolved) peptides within the acceptable margin of error.

## Introduction

MALDI-TOF is a convenient tool for determining peptide abundance in high-throughput workflows. MALDI-TOF MS is a t a solid-state ionization technique in which the sample is mixed with a chemical (matrix) that is excited by an ultraviolet or infrared laser. The laser excites the matrix leading to the transfer a proton to the analytes in the sample. The time of flight required for a given analyte to be detected is proportional to the mass of the analyte. The ions produced by this technique are primarily singly charged. Principles underlying each ionization technique have been well described elsewhere [1] Visualizing specific peptides or other analytes by exact mass allows for a greater degree of specificity in quantification and identification. In past work, MALDI-TOF has been used to measure angiotensin (Ang) peptides in cell culture or tissue samples and profile these peptides within their network [2]–[10]. Ang peptides belong to the renin angiotensin system (RAS), a hormonal system of major significance in human biology. The main effector of the system is Ang II [Ang-(1–8)], an octapeptide that is formed through sequential cleavage of the substrate angiotensinogen [11]. Among many other pathophysiological roles, Ang II is known to stimulate blood vessels to raise arterial blood pressure [12], activate mechanisms of sodium retention in the kidney [13] and induce proliferation in cardiac myocytes [14]. Interestingly, Ang peptides that are generated through alternative pathways of enzymatic processing, such as the heptapeptide Ang-(1–7), may elicit biological effects that are counteracting to those of Ang II [15]. Therefore, accurate visualization and quantification of Ang peptides is of utmost importance to adequately study the RAS. Proteins from tissue sections have been also analyzed, allowing for the localization of biological molecules to distinct regions of tissue [16]–[17], demonstrating the diversity and flexibility of MALDI-TOF analysis.

Mass spectrometry (MS) based techniques have advanced the field far beyond antibody-based methods with the capacity of identifying and quantifying multiple [18]–[20] peptides and posttranslational modifications in a single experiment [21]. The ability to quantify peptides is hindered by their physical and chemical properties. Differences in charge, hydrophobicity, or posttranslational modification are some of the properties that effect the ion formation and time of flight of a sample peptide or peptide mixture. Similar peptides can have widely varying differences in ionization within a sample, leading to differential matrix suppression or ‘flyability’ [2] between peptides. Flyability refers to the differences in ionization and post-source decay between similar peptides. Similar peptides may be more or less prone to ionize and therefore will generate a higher or lower signal, respectively. In quantification, flyability can be obtained through a constant based on known differences between peaks of different peptides [2].

Traditional methods of peptide quantification utilize the specific binding properties of antibodies to estimate abundance. Enzyme-linked immunosorbent assay (ELISA) and radioimmunoassay (RIA) are popular methods that indirectly measure the amount of bound antibody to the native peptide by a colorimetric reaction or radioactive decay [22]–[23]. One of the drawbacks of antibody-based methods is the potential for cross-reactivity with non-target peptides. Peptide quantification by mass spectrometry is direct, thereby avoiding issues associated with antibody cross-reactivity, and include those based on stable isotope dilution theory, although label free methods have been described [24]–[35]. Stable isotope dilution theory is based on the concept that a stable isotope labeled protein or peptide behaves exactly the same during MS analysis. Because the mass difference between the labeled and unlabeled samples can be detected through direct comparisons of signal intensities (*e.g.* time-of-flight) or area under extracted ion chromatograms (*e.g.* LC-MS/MS) are used for quantification. For the case of LC-MS/MS based quantification the sample is first separated prior to being introduced into the mass spectrometer thereby reducing the complexity and competition for ionization [31]–[34]. Due to lower complexity, the likelihood of overlapping peptide masses is also reduced and extraction of peptide specific fragment ion intensity over time can further increase the specificity of the measurement.

For monitoring biological reaction product formation, such as peptide metabolism, MALDI-TOF MS is ideally suited and takes advantage of the internal standardization commonly referred to as AQUA [35]–[38] (Absolute QUAntification of protein). Although MALDI-TOF does have limitations in its reproducibility due to the effects of uneven matrix-analyte mixture and matrix interactions, the use of SIS quantification allows for the circumvention of some of these difficulties. All SIS methods involve placing a known quantity of isotopically labeled peptide in a sample and comparing the peak intensities between the labeled and native peptide. The synthetic SIS peptide is identical to the native peptide with the exception that one amino acid is comprised of stable isotopes of carbon (^13^C) and nitrogen (^15^N). In practice both peptides are chemically identical with respect to ionization and decomposition, but the stable isotope labeled peptide is heavier and is detected as a different m/z window in the mass spectrometer thus allowing simultaneous comparison with the native. One or more amino acids can be labelled imparting further flexibility in the monitoring of peptide metabolism. The sum of the intensities of the first two or three peaks (M, M+1, or M, M+1, M+2) depending on the visibility of the peaks or the sum of the areas under the curve (AUC) of the peaks, calculated using Riemann sums or pixel counting, for a given peptide are divided by the same measure of the labeled peptide [36]. The peak intensity is defined as the maximum height of the peak. The Riemann sum AUC is the trapezoidal sum of the area under each peak. In both approaches, a cutoff or baseline is used to remove the effect of signal noise and is subtracted from the peak height or AUC. This ratio of native to labeled peptide is then multiplied by the known amount of the labeled peptide and sometimes corrected for response based on an external standard curve to estimate the amount of unmodified peptide.

This method of quantification is not without its difficulties; error in quantification can range from 2% to 12% [35]. This error has several possible sources from both methodology used for quantification and from the analysis itself. A significant amount of the sample can be lost during preparation due to manipulation before the addition of the labeled peptide. The amount of SIS peptide needed for accurate quantification can vary between experiments depending on the peak intensities found in the sample. The ratio between native and SIS peptide need to be less than 10 to aid in accurate estimation [38]. There is also the fact that there needs to be a SIS peptide for each peptide of interest in a sample to insure an accurate estimation of that peptide. This means, mixtures of SIS peptides should be balanced with endogenous levels for a given experiment. The need for multiple peptides and the quantity needed to fine tune the mixtures and preform the actual measurements begin to highlight the costs of peptide quantification by SIS peptides. In SIS quantification, the peptide(s) being quantified are known beforehand. This is necessary to produce the SIS version of the peptide, bypassing any problems that may occur by matrix suppression (or difference in ionization). The Gaussian mixture method incorporates the chemical properties of the known peptides by parametrizing the probability density function. The approach also provides discrete peak separation and provides the characteristics through the estimates of the unknown parameters of the isotopic distribution of the peptide being examined. An efficient algorithm for estimating the baseline is also incorporated into this approach. Unlike the existing Gaussian mixture approaches [38]–[43] by incorporating the known chemical information in the parameterization our approach reduces the dimensionality of the unknown parameter space. In addition to providing a more accurate quantification, the approach considerably speeds up the computations. In the convolved peptide situation peak intensity and Riemann sum AUC cannot be used to accurately quantify the separate peptides. Since this method can be automated over a large number of spectra and peptides, bottlenecks in the data processing pipeline are avoided.

Past methods are vulnerable to errors in data processing. The estimation of a baseline and cutoff regions for measurement are often end-user dependent or automated by proprietary software, both of which are often accepted and unquestioned. The calculation of the peak intensities (heights) or AUC are affected by changes in the signal-to-noise ratio and the resolution of the individual peaks within the spectra. Combine this with the problem of quantifying individual peptides of similar mass that form sets of overlapping peaks and even with SIS methods, quantification can become a difficult task using either of these signal intensity measures. Methods like liquid chromatography can be used to isolate convolved peptides but this adds additional sources of sample loss, are expensive in terms of both manpower and funding and do not scale easily to high-throughput workflows.

In the study of the renin-angiotensin system (RAS), the use of SIS methods for peptide quantification have been used to map out the extracellular processing of angiotensinogen prior to its biological action [2]–[9] at a given cellular target. Ang peptides from the RAS serve as good examples of isolated isotopic clusters (Ang-II: octopeptide, molecular weight (MW) 1046 Da) and convolved clusters (Ang-(2–10): nonapeptide, MW1081 Da and Ang-(1–9): nonapeptide, MW 1083 Da) detected by mass spectrometry.

While Gaussian mixtures have been used in the analysis of mass spectra [38]–[43], as mentioned earlier, these methods do not incorporate known characteristics, such as probable isotopic distribution of a compound, and have not been used to address the issues of peptide quantification. The proposed Gaussian mixture method takes into account the known physical constraints, such as isotopic mass separation and point distributions. The peak areas are estimated with the same error range as the peak intensity and peak AUC methods of SIS quantification in Ang peptide data. The range of error is carried over to convolved groups of peptides where no direct comparison of methods can be made. This method is easily automated using an R-package, for implementation of a multiple peptide search over several spectra.

Due to the abundance of data generated from MS analysis, there are several software packages that can be used to aid in the analysis of MS data. These include commercial packages such as Progenesis MALDI (Nonlinear USA Inc., Durham, NC) and many open platform packages that have been produced individually [44]–[49]. Each of these uses different methods for identifying and quantifying mass spectra, the details of which have not been published. Here, the aim is to show the versatility of partially known Gaussian mixture method in dealing with overlapping peptide clusters in the framework of SIS peptide quantification.

## Materials and Methods

The purpose of this study was to provide a new approach to the problem of quantifying single and convolved peptides in MALDI-TOF MS data using a Gaussian mixture model to measure and compare native peptides to SIS peptides. This approach will be compared to the established methods of single peptide quantification: peak intensity and Riemann sum AUC peak quantification for single peptide quantification. Peptides of the RAS were used for studying the characteristics of our approach and for comparing with other approaches.

### Mass Spectra Collection

Samples were examined using MALDI-TOF MS. Ratios of native and SIS peptides (Sigma-Aldrich, St. Louis, MO) were mixed in 2% aqueous trifluoroacetic acid (TFA). The SIS peptides are 6 Da larger than the native peptide as a result of [^13^C.^15^N]-valine incorporation into the amino-acid sequence. Concentrations of native and labeled peptides ranged from 20 to 1000 nmol/L (Table 1) depending on the ratio required. SIS-Ang-(1–9) has a MW of 1189.56, SIS-Ang-(2–10) a MW of 1187.71 and SIS-Ang-II a MW of 1052.59.

**Table 1:** Method Comparison Summary.

Method	MPE	MSE	Variance	Bias	95% CI
Gaussian Mixture	0.05172	0.00018	0.00201	0.05154	[0.03449, 0.06895]
Peak Intensity	0.05876	0.00030	0.00465	0.05845	[0.03255, 0.08497]
Riemann Sum	0.07691	0.00045	0.00598	0.07646	[0.04718, 0.10664]
Gaussian Mixture, Convolved Peptide	0.06801	0.00064	0.00264	0.06737	[0.03765, 0.09837]

The mean percent error (MPE), MSE, Variance and bias of each method’s percent error of the peptide ratio for various methods of ratio quantification. While all methods fall within the error parameters of the SIS method The Gaussian mixture model produces estimates in both single and convolved peptides while the peak intensity and Riemann sum methods of estimation cannot be used in convolved peptides.

Samples were applied to a MALDI target with a sandwich of α–cyano-4-hydroxycinnamic acid (cyano matrix) mixed in a one to one ratio (10 g/L) with 50% acetonitrile/0.1% TFA. The sandwich consisted of 2 µL of cyano matrix, 2 µL of sample, then another 2 µL of matrix. Each application was allowed to dry prior to the application of an additional layer. Spectra were collected in reflectron mode using a M@LDI MALDI-TOF mass spectrometer (Waters Corp., Milford, MA and AB SCIEX, Farmingham, MA). Twenty spectra were combined for analysis and were converted from MassLynx.raw directories to.mzXML files using MassWolf [48] or from.mgf to.mzXML using ProteoWizard [49] MSConvert for import into an R computing environment, version 3.01, [50] for analysis.

### Mass Spectra Data Processing

Once the data were imported into an R environment, the XCMS [51]–[53] package is used to load the .mzXML file and isolate the region around the known mass of the group of peptides of interest. This range is −1 m/z from the monoisotopic mass [M+H^+^] of the smallest peptide to +5 m/z from the monoisotopic mass of the largest peptide in the group. These ranges of peptide masses were grouped together based on the overlap of isotopic clusters of individual peptides within the above range. Peak area estimation was performed by constructing a Gaussian mixture model for each peptide.

The Gaussian mixture is a multimodal distribution the density of which is produced by a weighted sum of Gaussian densities. In the MS context, the density could be written
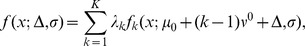
where 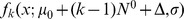 is the Gaussian density with mean 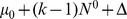 and variance 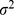 for the *k*th component of the Gaussian mixture, 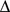 is the mass error (or accuracy) of the spectra due to error in the standard curve calibration of the mass spectra, 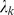 is the proportion of the *k*th component as defined by the isotopic distribution of the peptide that is limited by the total amount of the peptide accounted for, 99.99% in most instances, 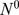 is the mass of a neutron (1.00866912 Da). The square root of the variance, namely the standard deviation, 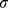, could also be interpreted as the peak width. Peak resolution can be obtained from the standard deviation by simple transformation of the ‘Full-Width Half-Maximum’ of the peak and it can be explained in terms of 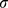 as 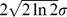. Although general Gaussian mixtures allow for the variance of each component Gaussian density to vary, since the resolution of individual peaks in MALDI-TOF are equal [1] with the change in variance between peaks increasing (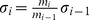), it is reasonable to set the variance across the examined m/z range to be equal since the change in variance is very small over the range being examined. The mass difference between peaks of a single peptide is 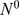. The addition due to this mass is negligible over the small m/z range seen in SIS quantification. For clusters of peptides, the Gaussian mixture of each peptide is combined across the mass range without additional weighing of the individual peptides. The peptide peak areas associated with the mass error and peak width (namely 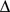 and 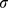) yielding the best fit is used as the area estimates for that cluster of peptides. Goodness of fit of the model is determined by the *R* ^2^, the coefficient of determination (computed as the average of the squared distance between the observed and estimated peaks). The *R* ^2^ is calculated over a range of mass error (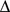) and peak width (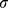) values and the area corresponding to the combination of 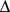 and 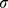 that yields the highest *R* ^2^ is considered the final estimate of the peak area.

**Figure 1:**
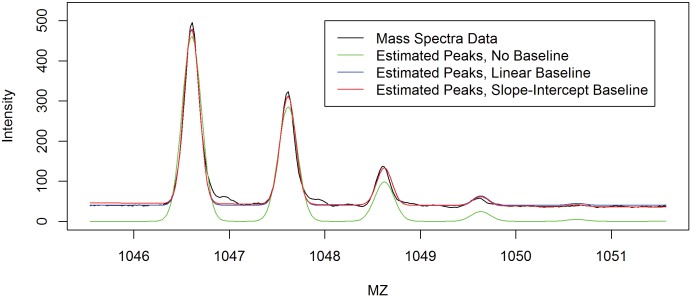
Different methods used for fitting a single peptide isotopic cluster to a MALDI-TOF spectrum of unlabeled Angiotensin II. The inclusion of a slope-intercept form baseline (red estimation) increases the fit over a flat baseline (blue estimation). Both of which are better than not including a baseline (green estimation).

**Figure 2:**
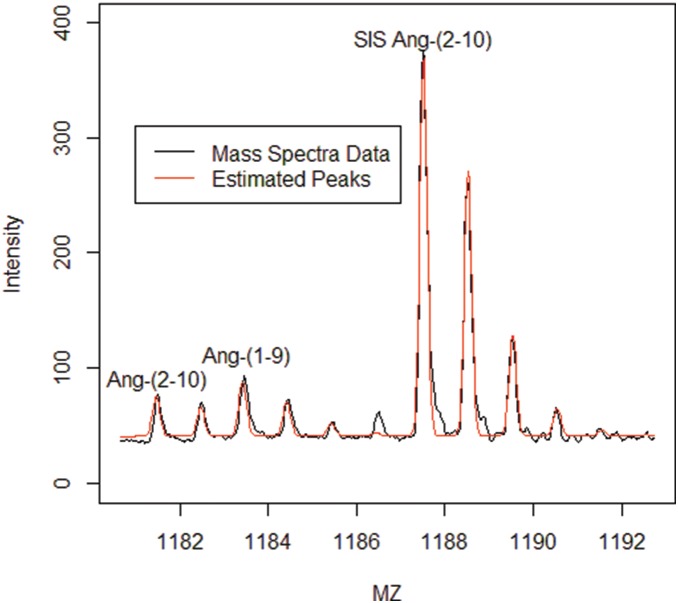
A MALDI-TOF mass spectrum from the analysis of Ang I extracellular breakdown [2] by rat glomeruli in the presence of amastatin (APA inhibitor) and thiorphan (NEP inhibitor) at 60 minutes. The sample contains a mixture of Ang-(2–10), Ang-(1–9), and SIS-Ang-(2–10) that overlap forming one cluster. These peaks are fit and the individual areas for each isotopic cluster can be decomposed from the spectrum.

**Figure 3:**
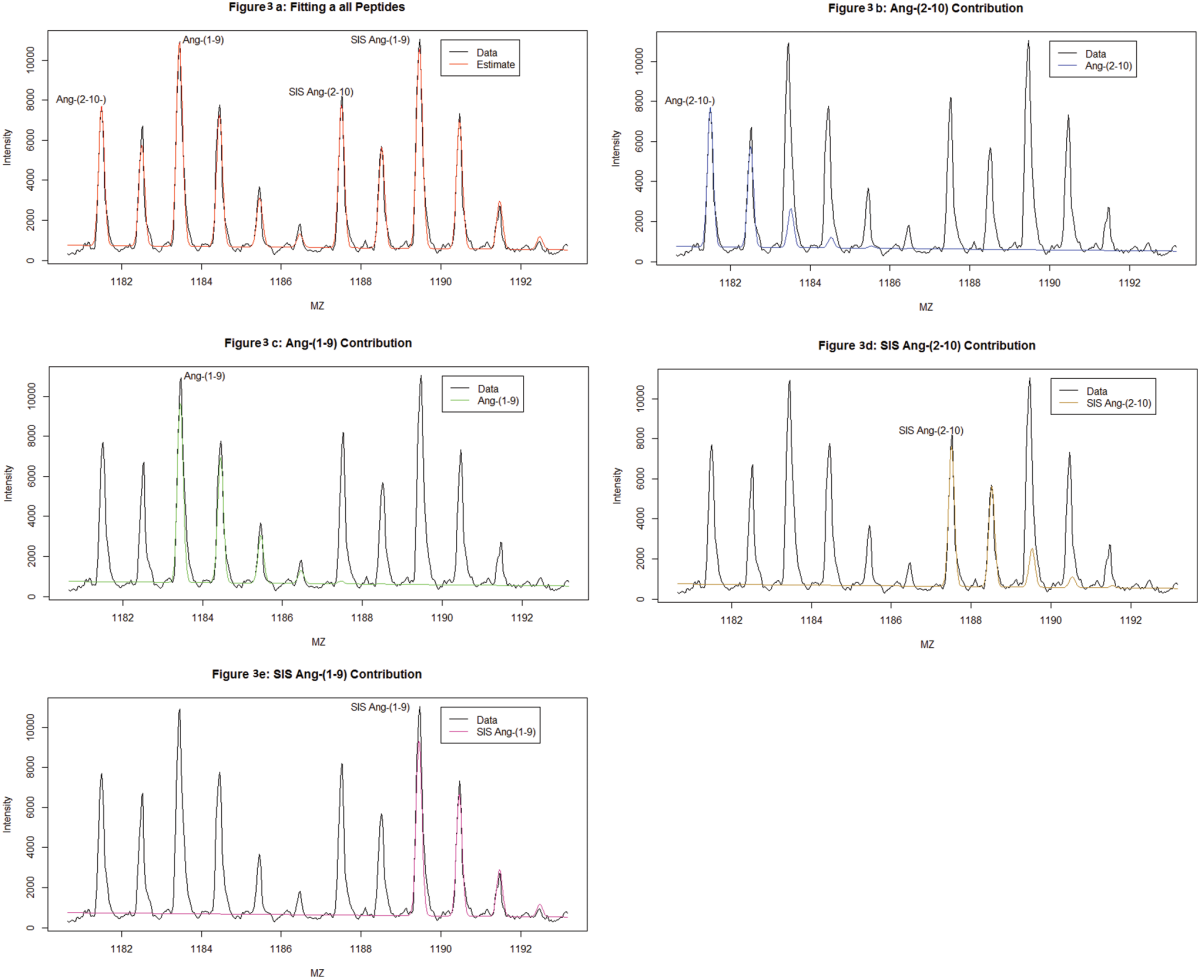
A MALDI-TOF mass spectrum of a known ratio of 1∶1∶1∶1 peptides consisting of 300 nM Ang-(2–10), Ang-(1–9), SIS-Ang-(2–10) and SIS-Ang-(1–9). The spectrum has been fit using GMM and the figure shows how each estimated peptides contributes to the whole spectrum. Since all peptides are estimated simultaneously, each peptide is presented here separately to illustrate the individual contribution of each peptide to the spectrum as a whole. (A) Figure 3a shows the entire estimation as a whole, preformed as a single fit to a single cluster of four overlapping peptides. The data is shown in black with the estimated peaks superimposed in red. (B) Figure 3b shows the estimated contribution of Ang-(2–10) to the spectra superimposed in blue. (C) Figure 3c shows the estimated contribution of Ang-(1–9) to the spectra superimposed in green. (D) Figure 3d shows the estimated contribution of SIS-Ang-(2–10) to the spectra superimposed in dark yellow. (E) Figure 3e shows the estimated contribution of SIS-Ang-(1–9) to the spectra superimposed in dark purple.

**Figure 4:**
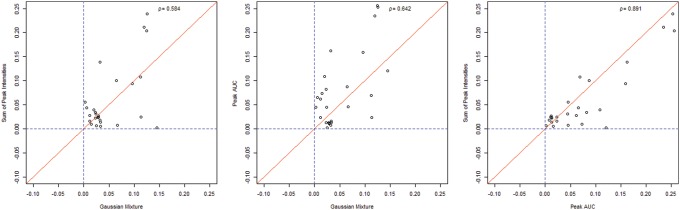
Correlation plots showing the difference in estimation error of the peak ratio for a given spectrum when different methods of peak ratio measurement. The red line denotes a correlation of ρ = 1 and the blue lines denote 0% error in ratio estimation for that given method. Here we see that the Peak intensity and Riemann sum AUC methods of quantification correlate more highly with one another than with the Gaussian mixture method. Note that the GMM estimates tend to cluster closer to the blue line suggesting lower error.

Examples of model fits using the Gaussian mixture are shown in Figures 1, 2 and 3 where the fit of a single peptide (Ang II, Figure 1) and a convolved set of peptides (Ang-(2–10) and Ang-(1–9) and SIS peptides, Figure 2 and 3). The estimated individual contribution of each peptide can be seen in in Figure 3. Often a vertical shift is observed in spectra and needs to be accounted for in SIS estimations [54]–[55]. This shift, hence forth called baseline shift, could be caused by many sources one of which might be the noise from the MS signal. It is believed that this noise could act in either an additive or masking (overlapping) fashion. There is no universally accepted method in the literature for estimating or correcting for this baseline shift. However, generally this error seems to be treated as additive and is treated as such with all methods discussed in this paper. The baseline shift is described here as a slope-intercept form of a linear function over the m/z range examined for each peptide cluster. Figure 1 is presented to illustrate the importance of including the baseline correction in the quantification. For this example, there is a less adequate fit (*R* ^2^ = .64) when no baseline is assumed, and the fit improves as the baseline is estimated, which improves further changed from a simple vertical shift (*R* ^2^ = .97) to a shift on a gradient (*R* ^2^ = .99) described as a slope intercept equation. Figures 2 and 4 shows the fit of a convolved peptide model (*R* ^2^ = .94) using a slope intercept baseline shift.

The proposed approach estimates the mass error and peak width (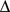, 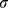 respectively), over a range decided by the researcher. Then peak area for each individual peptide and the slope intercept of the baseline shift are simultaneously estimated using a QR decomposition of a linear model between each individual peptide, the intercept, and the m/z range the cluster covers, and the spectrum data over that same m/z range. Then the mass and isotropic distribution based on the composition of each labeled and native peptide is estimated. Peptides with masses within 5 DA of each other are then convolved to estimate their abundance. (An R [49] package implementing this algorithm to estimate the mass error, peak width, and peak area has been written and will be made available subsequently.) A full workflow for the Gaussian mixture method algorithm as described is shown in figure S1.

Two currently available methods for SIS quantification are considered for comparison against the Gaussian mixture method. The methods are the peak intensity measure and the Riemann sum AUC methods of quantification. The peak intensity measures the height of the identifiable peaks in a given isotopic cluster and sums them. The Riemann sum AUC is trapezoidal sum of the area under the identifiable peaks. For single peptide clusters, the peak intensities of the visible major peaks (usually monoisotopic, M+1, M+2) and a Riemann sum of the major peaks were estimated for comparison (Table 1). Both peak intensity and Riemann sum methods require a baseline shift correction, which are often made using one of several methods in the literature [26]–[27], [44]–[47], [54]–[55]. (For the comparisons made in this manuscript, the baseline shift estimated from the Gaussian method will be used).

Theoretically, the expected locations of the peaks are known to be at the mass for each peak (M, M+1, M+2) shifted by the error adjustment. The new masses (M+Δ, M+1+Δ, M+2+Δ) are each then used to center a 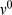 range on the mass-to-charge ratio axis (MZ). The peak intensities within this range are searched for the maximum intensity. Once the estimated baseline at this MZ is found, it is subtracted from the overall intensity at this point and this is used as the peak intensity for that peak. Riemann sums are calculated over these same three MZ ranges using the formula
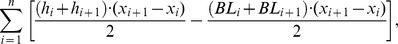
where *h_i_* is the intensity of the spectrum at point x_i_ on the MZ axis and BL is the baseline estimate used for noise subtraction at this point on the MZ axis.

Once the peak areas for the best fitting model are collected the ratios of native to labeled peptide are calculated form the peak intensity, Riemann sum areas and Gaussian mixture areas. (Table 1) Since peak intensity and Riemann sums cannot be estimated for all individual peptides in overlapping isotopic clusters of peptides, only the Gaussian mixture method estimates are obtained for overlapping peptides.

Once ratios were calculated for the various measures of signal intensity for the pairs of native and labeled peptides these were used to calculate percent error form the know ratio present in the sample. The absolute difference between the known and estimated ration was divided by the known ratio. These percent errors in ratio estimation were then used to compare between samples and peptides. This was done to limit the interference inherent in samples with a range of signal intensities.

### Statistical Analysis

To compare the various methods, the fits are expressed as percent error of a given ratio and the mean, mean square error (MSE), variance and bias for each of the three methods are presented. (Table 1, Table S1) Scatter plots of the percent error of the true ratio between methods for each spectra analyzed (n = 26, Material S2) will be presented for graphical illustration of the agreement between methods (Figure 4). A two-way ANOVA with peptide (Ang II and Ang-(2–10)) and method (Peak Intensity, Riemann Sum AUC and Gaussian mixture model) as independent factors and adjusting for the subsampling (sample replicates) by including a random effects term in the model will be used to formally test the null-hypothesis that the methods are similar. From this analysis the least squares estimates of the mean for each method, along with post-hoc confidence intervals adjusted using Tukey’s approach will be presented (Table 2).

**Table 2:** Two way ANOVA with pairwise testing.

Method	Mean Estimate	Pr>|t|	95% CI
Peak Sum	0.07181	<0.0001	[0.049, 0.095]
AUC Sum	0.08999	<0.0001	[0.067, 0.113]
Gaussian	0.05562	<0.0001	[0.032, 0.079]
**Pairwise comparison of means**			
**Method**	**Mean Difference**	**Pr>|t|**	**95% CI**
Peak Sum v. AUC Sum	−0.01818	0.2712	[−0.051, 0.015]
Peak Sum v. Gaussian	0.01619	0.3270	[−0.017, 0.049]
AUC Sum v. Gaussian	0.03437	0.0399	[0.002, 0.067]

The Two-way ANOVA analysis takes into account that several samples are replicates of a single mixture of peptides (Material S1) and that there may be differences between peptides used and not just the methods of peak quantifcation. All standard errors for the mean estimates were equal (0.01159).

Since the currently available methods are not applicable for the estimation of convolved peptides, there cannot be a direct comparison between the above methods using convolved peptide data. An analysis of mean, mean square error (MSE), variance and bias was used to compare single and convolved peptide estimation using the Gaussian mixture method (Table 1, Table S2).

## Results

### Single Peak Analysis

As a proof of principle, the Peak Intensity and Riemann sum AUC methods of signal measure and the Gaussian mixture method were used to examine 26 spectra (9 Ang-(2–10)/SIS-Ang-(2–10) and 17 Ang-(1–9)/SIS-Ang-(1–9)) that consisted of replicate MALDI-TOF analysis of seven different mixtures. These measures were then used to back calculate a ratio of native to labeled peptide which was then compared to the true ratio. Since varying ratios were involved, the percent error of the true ratio was used to measure predictive capacity of all three methods. Peak intensity and Riemann sum used the first three visible peaks for comparison (M, M+1, M+2). The Peak Intensity method was found to have a mean error of estimation of 5.9 [3.3, 8.5]%. The Riemann sum method was found to have a mean error of estimation of 7.7 [4.7, 10.7]%. The Gaussian mixture method was found to have a mean error of estimation of 5.2 [3.4, 6.9]% (Table 1). The mean errors seem to fall within the range of accepted SIS accuracy [34] and share low variances across all three methods.

Correlation plots between methods show that the peak intensity measure and Riemann sum are highly correlated (ρ = 0.89) and that the Gaussian mixture method is similarly correlated (ρ = 0.58, 0.64) with the other methods implemented (Figure 4).

The two-way random effects ANOVA gives more accurate estimates (Least Square Means) for the comparison of the methods (Table 2). The estimated means of the methods are 7.2 [4.9, 9.5]% for Peak Intensity, 9.0 [6.7, 11.3]% for Riemann sum and 5.6 [3.2, 7.9]% for Gaussian mixture. The difference between Peak Intensity and Riemann sum was not significant and the difference between Peak Intensity and Gaussian was not significant (p>0.05), but the difference between Riemann sum and Gaussian methods was significant (p<0.04).

The analyses of different ratios of Ang-(2–10) and Ang-(1–9) that were considered are summarized in Table 3. Ten samples of the three ratios were used for a total of 30 spectra. The 2–10 was increased while 1–9 and both SIS peptides were kept constant. The mean error estimate was 2.97 [2.5,3.4]% for 1∶1 ratio, 5.7 [.57,10.7]% for a 2∶1 ratio, and 5.3 [4.2,6.4]% for a 10∶1 ratio.

**Table 3:** Secondary peptide ratio estimation.

2–10∶1–9 Ratio	MPE	MSE	Variance	Bias	95% CI
1∶1	0.02971	0.00100	0.00013	−0.02146	[0.0254, 0.03401]
2∶1	0.05684	0.01975	0.01835	−0.05555	[0.00574, 0.10795]
10∶1	0.05315	0.00361	0.00087	−0.02585	[0.04201, 0.06428]

Here the Gaussian mixture method is used to recover the peptide ratio of the second peptide in a convolved set. The error in the estimation of an Ang-(1–9) peak ratio against its corresponding SIS peptide when convolved with various amounts of Ang-(2–10) and its corresponding SIS is used to test the Gaussian mixture method. The initial concentration of 300 nM of each peptide (for a 1∶1∶1∶1 ratio of peptides, Material S2) is modified by changing the amount of Ang-(2–10). Here we see that the Gaussian mixture method can recover the second peptide from a series of different peptide ratios.

### Convolved Peak Analysis

Convolved peaks are formed by overlapping ionic currents as described earlier. An example of a typical convolved peak problem consisting of multiple peptides is shown in figure 2. The Gaussian mixture method is the only method capable of decomposing convolved peptides. To examine how well the Gaussian mixture method can be used to estimate peptide ratios for this type of quantification, eleven spectra representing convolved peptides (5 replicate Ang-(2–10)/SIS-Ang-(2–10)/SIS-Ang-(1–9) and 6 replicate Ang-(2–10)/Ang-(1–9)/SIS-Ang-(2–10)/SIS-Ang-(1–9)) were analyzed. The Ang-(2–10) ratios and Ang-(1–9) ratios were calculated and compared to the true ratios. The mean error of estimation from these eleven spectra was found to be 6.8 [3.8, 9.8]%.

## Discussion

Our findings demonstrate, the Gaussian mixture method is capable of handling both single and convolved peptides for the estimation of SIS ratios with similar accuracy but the performance of the method is sensitive to peak resolution and signal to noise ratio. The single peptide estimations produce similar (or better) results compared to the two previously used methods. For convolved peaks, the Gaussian mixture method produced similarly accurate results, while previous two methods treat those situations intractable. All of the means fall within the acceptable levels [18], of error for SIS quantification and provide a basis for the equivalence of the results from the Gaussian mixture model method for estimating convolved and non-convolved peptides. Gaussian mixture method is more advantageous because it can be applied to both single peptide and multiple, overlapping peptides with at least the same accuracy as past methods. It also supplies a mathematical justification for baseline estimations instead of an *ad hoc* approach.

There are a few limitations in using all the three methods and some are specific to the Gaussian mixture. A closer examination of the correlation plot (Figure 4) reveals a grouping of points that seem to be outliers. These points that cluster furthest from the diagonal represent samples that on closer examination had lower resolution and/or exhibited skewed peaks. This cluster of three data points is farther from the main cluster of data, suggesting a poor estimation of the ratio using all methods of peak quantification. The ability to calculate the native:SIS ratio is affected by the quality of the data being examined. Quality can be quantified by the resolution (or variance of the component normal of the Gaussian mixture distribution) of the peaks. Low quality (high variance, low resolution and/or misshapen) peaks are harder to quantify using the Gaussian mixture method. In other words, if the underlying assumption of normality under each peak is violated the Gaussian mixture method might produce larger errors. The Gaussian method is more sensitive to the resolution, returning higher error ratio estimates with the lower resolution spectra than previous methods. The Gaussian mixture method does predict ratios with better accuracy with higher resolution spectra than previous methods. This needs to be explored further by analysis of signal to noise ratios and there correlation with resolution. It is anticipated that the higher resolution will produce larger signal to noise ratios, which would explain the sensitivity. This method is dependent on knowing the exact mass of peptides being quantified in a given sample. Because GMM data derived from MALDI-ToF alone analyzes only the intact charged mass, not reacted to produce highly specific fragment ions (e.g. b/y ions) like that for other mass spectrometry modalities, unknown compounds that are nearly identical in mass can confound the accuracy of the measurement. Only the highest resolution instruments, such as MALDI- Fourier transform ion cyclotron resonance mass spectrometers, can achieve peak resolution that can minimize this overlap. Furthermore, due to their low abundance in plasma, enrichment strategies are often necessary to measure vasocactive peptides by MALDI-TOF [29], [56] which is a low sensitivity detection system in the presence of a high matrix environment. In experiments attempting to profile the metabolism of vasoactive peptides and quantify the end-products, where GMM is most relevant, requires the addition of an exogenous peptide to a high concentration necessary to elevate the signal to detectable levels [2]–[5], [7], [8], [10]. Whether or not GMM is applicable to the analysis of native biological samples is likely to require testing on a case by case basis using high resolution instruments prior to the use of MALDI-ToF alone.

The error in estimation increases for convolved peaks (compared to single peak estimation errors which ranged between 0.051% and 0.068% (Table 1). The analysis of Ang-(2–10) with a convolved set of SIS peptides shows that convolved peaks when decomposed can be estimated within the same error range as single peptide peaks but sets of convolved peptides (Figure 2,3) show an increase in the error of estimation (Material S1). This estimation error may be corrected in MALDI-TOF data by adjusting the peak width estimation by a correction based on the static resolution of the data. This will be explored in future research. Even with these increased errors in estimation of the convolved peptide ratios, the ratios are estimated within the same allowable error range [35]. It is possible that additional mixture parameters, such as a variable peak sigma that narrowly increases across peptides’ m/z range or the use of flyability constant as in previous work [2] may need to be incorporated to estimate multiple sets of convolved peptides.

Future refinement of the Gaussian mixture method will require the examination of several aspects of the algorithm. The appropriate cutoff for the number of isotopic peaks that constitutes the significant majority of the peptide in the sample also affects the minimum m/z that needs to be considered for calling two peptides separate. This defines their status as a convolved cluster or as peptides to be considered individually. This is expected to be a function of the atomic composition of a given compound, where the more atoms comprising the molecule lead to a larger and more complex isotopic distribution. Methods for adjusting estimations of peptide isotopic distributions that reflect possible local variations will need to be considered to see if they are viable and make a significant addition to area estimations. Implementation of a maximum likelihood estimator of the Gaussian parameters will increase both speed and accuracy of this method, but other measures of ‘goodness of fit’ need to be explored. Implementation of a quadrant search algorithm for exploring the parameter space needs to be implemented to accelerate peak quantification for larger data sets. Finally, simulation studies are required to validate this method over a wide range of extremes in spectra composition. Such a study is being considered and will appear in a subsequent publication.

The use of informed Gaussian mixture method is a novel approach to peptide quantification with the tangible benefits of the flexibility to tackle traditional single peptide cases and overlapping peptides as well. It also provides baseline estimation with mathematical justification. This process can also be automated for multiple peptides over multiple spectra allowing for a high through put quantification analysis. The Gaussian mixture method is comparable to both Peak Intensity and Riemann sum methods of signal measure in SIS quantification. When dealing with convolved peptides we show similar levels of error relative to non-convolved peptide area and ratio estimates with the Gaussian method. The Gaussian method is equivalent, will remove the *ad hoc* baseline estimations used else-where, and will give estimations that fall within the range of acceptable SIS error for both convolved and non-convolved peptides. This method could be implemented in a reasonable amount of time for quantification of any compound, with known composition, examined using mass spectrometry and an internal standard. The use of the Gaussian mixture is also variable since mixtures of other distributions could be used to better describe other spectra where necessary.

## Supporting Information

Figure S1 **A short workflow of the GMM algorithm used to fit MALDI-TOF MS data.**
(TIF)

Table S1 **Contains the raw fit results from the GMM analysis of data in materials S1 (single peptide spectra), an expanded LS means table, the results of the peak intensity and Riemann sum analysis of the single peptide samples, and individual peptide ratio calculations.**
(ZIP)

Table S2 **Contains the raw fit results from the GMM analysis of data in materials S2 (convolved peptide data).**
(CSV)

Material S1 **Single peptide spectra data.**
(CSV)

Material S2 **Convolved peptide spectra data.**
(CSV)
