# Supplementary figures and images for: The Application of Gaussian Mixture Models for Signal Quantification in MALDI-ToF Mass Spectrometry of Peptides
Source: PLoS One. 2014 Nov 5;9(11):e111016. doi: 10.1371/journal.pone.0111016 (PMC4221630; doi:10.1371/journal.pone.0111016)

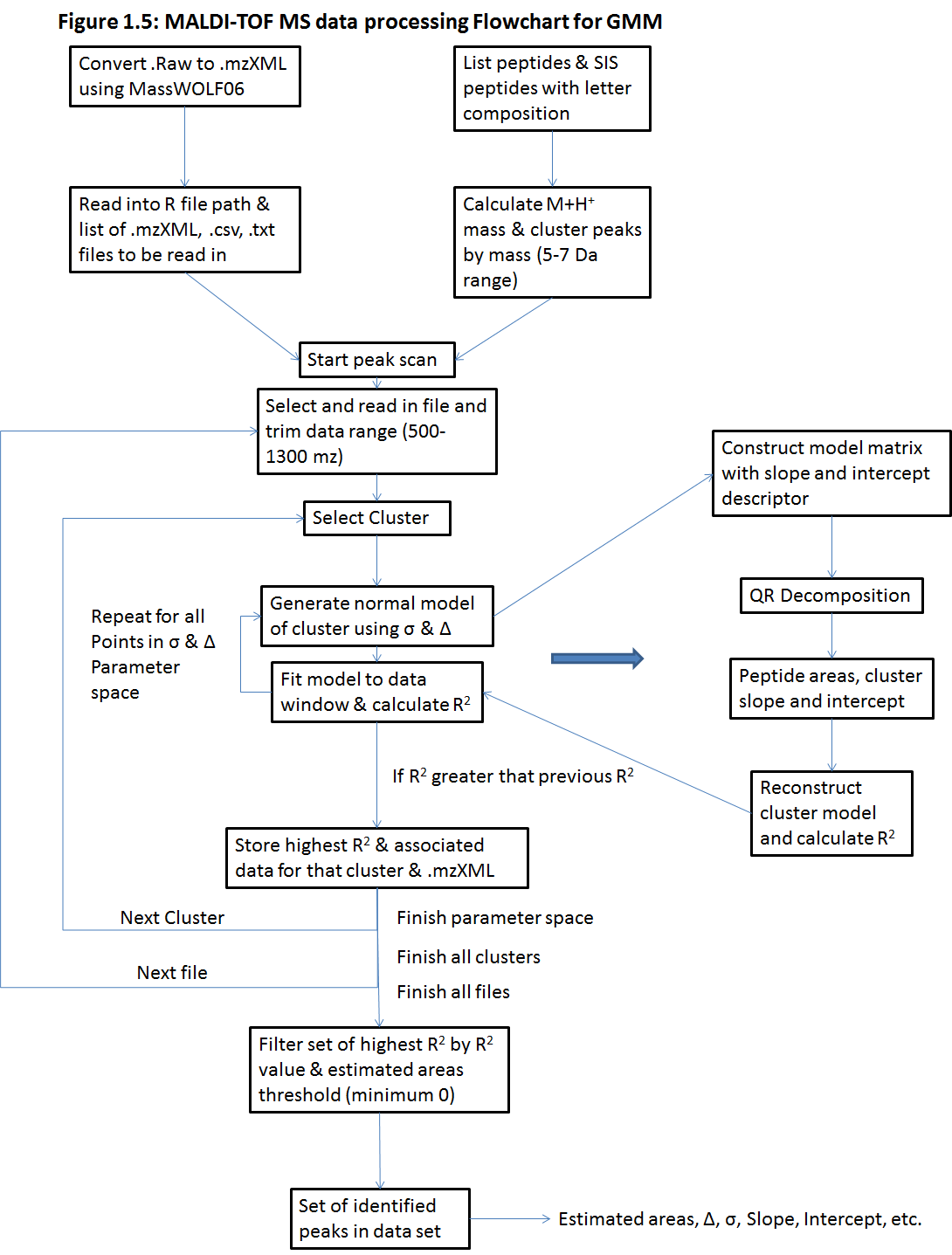

Supplement: Figure S1 — A short workflow of the GMM algorithm used to fit MALDI-TOF MS data. (TIF) [file pone.0111016.s001.tif]
